# Serving the Underserved in an Underserved Setting

**DOI:** 10.1002/alz.092529

**Published:** 2025-01-09

**Authors:** Ayda K Tefera, Lea T. Grinberg

**Affiliations:** ^1^ Global Brain Health Institute, San Francisco, CA USA; ^2^ Eka Kotebe General Hospital, Addis Ababa Ethiopia; ^3^ Global Brain Health Institute, University of California, San Francisco, CA USA; ^4^ Memory and Aging Center, UCSF Weill Institute for Neurosciences, University of California, San Francisco, San Francisco, CA USA

## Abstract

**Background:**

The COVID‐19 pandemic in Ethiopia posed additional challenges to an already strained mental health service. Eka Kotebe Hospital, the second‐largest mental health facility with a capacity of 175 beds, was transformed into a dedicated COVID‐19 treatment center, leaving mental health service users, especially vulnerable elderly patients with cognitive impairments, without adequate support.

I had the challenge to implement alternatives to provide mental health services coverage to underserved elderly population. Here, I describe the process and results of a novel community outreach project done in partnership with a local nursing home (Mekedonia) which shelters homeless elderly and people with mental illness.

**Method:**

Following the formalization of MOU, our master’s level professionals and psychiatric nurses, started to conduct four visits per week to Mekedonia. The primary goal was to identify complex cases and tailor management plan.

**Results:**

At the program inception, the shelter housed approximately 5000 individuals, necessitating an initial task of conducting comprehensive health assessments and classifying patients based on their clinical conditions. Most of the residents in the shelter are persons with intellectual disabilities, elderly individuals with mental illness and dementia.

The ongoing pandemic further compounded the difficulties in conducting thorough assessments, with majority of assessments relied heavily on clinical history, which was inaccurate due to lack of knowledgeable informant, and Mini‐Mental State Examination (MMSE).

Remarkably, a significant portion of the clients had their first encounter with a medical personnel through our program, revealing a high prevalence of multiple medical comorbidity among this population. Management includes controlling aggression, treatment of insomnia, addressing comorbidity and facilitating referrals. The data indicated that most clients were 40 and above, In 2021 we were able to identify and manage 50 dementia patients and the number has doubled to 100 in 2022 and 153 cases people were identified in 2023. Currently the program has sustained on bigger scale, and we are deploying GPs, Neurologists and Psychiatrists to address the diverse health needs of this vulnerable population.

**Conclusion:**

Despite all the challenges, the project underscored the importance of tailored outreach initiatives to address the unique needs of homeless elderly individuals during unprecedented times.

